# Effects of Wet Oxidation Process on Biochar Surface in Acid and Alkaline Soil Environments

**DOI:** 10.3390/ma11122362

**Published:** 2018-11-23

**Authors:** Qinya Fan, Liqiang Cui, Guixiang Quan, Sanfei Wang, Jianxiong Sun, Xiangyun Han, Jia Wang, Jinlong Yan

**Affiliations:** 1 School of the Environment and Safety Engineering, Jiangsu University, Zhenjiang 212013, China; QyFan0518@163.com; 2 School of Environmental Science and Engineering, Yancheng Institute of Technology, Yancheng 224051, China; lqcui8@hotmail.com (L.C.); qgx@ycit.cn (G.Q.); 18862063729@163.com (S.W.); 18762398866@163.com (J.S.); hxy16_2000@163.com (X.H.); wj1511701011@163.com (J.W.)

**Keywords:** biochar, oxidation, surface characteristics, functional groups, O/C

## Abstract

Biochar has been studied for remediation of heavy metal-contaminated soils by many researchers. When in external conditions, biochar in soils ages, which can transform its structural properties and adsorption capacity. This study was conducted with two oxidation processes, HNO_3_/H_2_SO_4_ and NaOH/H_2_O_2_, to simulate the effects of biochar in acid and alkaline soil conditions. The results show that the oxygen-containing functional groups increased in aged biochar, which led to improve the ratio of oxygen and carbon (O/C). Nitro functional groups were found in the acid-oxidation treated biochar. Destroyed ditches and scars were observed on the surface of aged biochar and resulted in growth in their specific surface area and porosity. Specific surface area increased by 21.1%, 164.9%, and 63.0% for reed-derived biochar treated with water washing, acid oxidation, and basic oxidation, respectively. Greater peaks in the Fourier Transform Infrared Spectroscopy (FTIR) results were found in C–O and O–H on the surface of field-aged biochar. Meanwhile, mappings of energy-dispersive spectroscopy showed that biochar aged in soil was abundant in minerals such as silicon, iron, aluminum, and magnesium. In summary, biochar subjected to wet oxidation aging had an increased capacity to immobilize Cd compared to unaged biochar, and the adsorption capacity of oxidized biochar increased by 28.4% and 13.15% compared to unaged biochar due to improvements in porosity and an increase in functional groups.

## 1. Introduction

With the pursuit of eco-friendly materials, people have shown great interest in biochar in recent years. Biochar is the product of oxygen-limited biomass pyrolyzed treatment [1,2]. Many studies have focused on biochar for soil remediation because of its specific physicochemical properties, such as complicated pore structure and the abundance of functional groups on its surface. Meanwhile, biochar meets the requirements of being a relatively inexpensive material in soil remediation [3,4,5,6,7]. Karhu et al. [8] spent 1.5 months analyzing an agricultural field that had been treated with 9 t ha^−1^ of biochar, and the results showed that soil remediated with biochar increased the uptake of CH_4_ by 96% and the water-holding capacity by 11%. Water-holding capacity is the most important factor in sandy soil remediation. Cui et al. [9] conducted an adsorption experiment (involving cadmium and lead) with peanut hull-derived biochar and found that the adsorbed cadmium (Cd^2+^) and lead (Pb^2+^) were mainly bound with carbonate (50%) and organic compounds (40%) from biochar. To enhance the adsorption of biochar, Diao et al. [10] immobilized nanoscale zero-valent iron on sewage sludge-derived biochar. According to the simulated removal of chromium (Cr^6+^) and lead (Pb^2+^), 90% Cr^6+^ and 82% Pb^2+^ were removed in 30 min by the nanoscale zero-valent iron immobilized biochar.

Recently, some studies have turned to the aging process of biochar in fields to evaluate whether biochar is a sustainable remediator. Kaudal and Weatherley [11] produced biochar by pyrolyzing a mixture of biosolids and green waste (2:1) at 650 °C. Then artificial aging was conducted by composting biochar with food waste (10% *v*/*v*) for 75 days to achieve something similar to terra preta. The results showed that co-composted biochar had a higher cation exchange capacity (CEC), pH, electrical conductance (EC), and nitrogen-loading capacity than uncomposted biochar. Paetsch et al. [12] compared the effects of in situ aged biochar and fresh biochar on water-holding capacity and microbial parameters in grassland soil. The comparison proved that biochar can ameliorate the water regime of soil, and the effect will be greater in drought conditions. Meanwhile, aged biochar increased soil organic carbon mineralization, which can improve carbon use in fields. Mia et al. [13] examined the effects of fresh chemical-aged and field-aged biochar on biological nitrogen fixation in soil. They claimed that fresh biochar decreased the fraction of symbiotic nitrogen fixation from 73% to 68%. Field-aged biochar reduced nitrogen fixation, but chemical-aged biochar did not show changes.

Applying biochar to fields as a soil remediator has been researched in many studies, but few of them take ground conditions into consideration. Here, we oxidized biochars pyrolyzed from different materials by two methods to simulate biochar application in different soil conditions. The first method was to simulate acidic soil affected by acid rain. In China, up to 30% of the area suffers from acid rain every year, leading to depressed crop production. The other was to simulate saline–alkali soil conditions. Biochar is an appropriate material to improve water-holding capacity and nutriments in saline–alkali soil, but the effects of basic conditions on biochar remains to be researched. This paper focuses on the effects of acid and basic oxidation of biochars made from different materials with regards to the biochar aging process in various environments and the changes present on the biochar surface.

## 2. Materials and Methods

### 2.1. Materials

Three different biochars were used in this study. Two biochars, produced from rice straw (RSB) and reed (RB), were pyrolyzed at 450 °C for 120 min with limited oxygen in our laboratory. Wheat straw-derived biochar (WSB) was also pyrolyzed at 450 °C, but we bought it from the Sanli New Energy Company, Henan Province, China, instead of pyrolyzing in the laboratory because we needed a large amount for the field experiment. The basic physicochemical properties are presented in Table 1.

### 2.2. Experimental Design

All biochars were pretreated before oxidation. The biochar was put into deionized water (*m*/*v* = 1/100) and shaken at 60 °C for 12 h in a temperature-controlled water bath. After that, the biochar was separated with 0.45 μm glass fiber filter paper (Kejia, Shandong, China) and dried in an oven (Yiheng, Shanghai, China) at 105 °C for 4 h. Pretreated biochars were labeled pretreated WSB (PWSB), pretreated RSB (PRSB), and pretreated RB (PRB).

NO_3_^−^ and SO_4_^2−^ are the most common acidic ions in acid rain, so we simulated the effects of acid rain on biochar in the field by adding HNO_3_/H_2_SO_4_ (*v*/*v* = 1/3); 2.5 g of pretreated biochar was added to 200 mL of a 20% HNO_3_/H_2_SO_4_ mixture. After shaking for 6 h, the mixture was separated with 0.45 μm glass fiber filter paper. The oxidized biochars were washed with deionized water until the pH of percolate was stabilized [14,15]. Finally, all biochars were dried as described above and labeled acid WSB (AWSB), acid RSB (ARSB), and acid RB (ARB).

Saline–alkali soil is also a remediating target of biochar. An NaOH/H_2_O_2_ mixture (50 mL of 1.0 mol L^−1^ NaOH and 10 mL of 30% H_2_O_2_) was used to create the basic conditions, and 20 g of pretreated biochar was mixed with 50 mL of 1.0 mol L^−1^ NaOH and 10 mL of 30% H_2_O_2_ in polyethylene bottles. After standing for 30 min, the bottles were capped and shaken for 24 h, then they underwent the same separation as described above. The oxidized biochars were dried and labeled basic WSB (BWSB), basic RSB (BRSB), and basic RB (BRB).

Meanwhile, the biochar that was applied to the field (31°24.434′ N and 119°41.605′ E) with 20 t ha^−1^ 8 years ago (2009) was separated for comparison with the simulated oxidation. The biochar was separated by centrifuging (Feige, Shanghai, China) the light fraction after suspending the soil in 1.6 × 10^6^ g m^−3^ sodium polytungstate solution for 30 min [16,17]. The sample was dried and labeled FWSB.

Adsorption kinetics and isotherms were also conducted in this study. First, 0.1 g of biochar was mixed with 50 mL of 40 mg g^−1^ Cd solution and shaken for 5, 15, 30, 60, 120, and 480 min. Adsorption isotherms were obtained by putting 0.1 g biochar into 50 mL solutions whose initial Cd concentrations varied from 5 to 40 mg L^−1^. All samples were filtered and measured by atomic absorption spectroscopy (Zeenit 700p, Jena, Germany).

### 2.3. Analytical Methods

The elemental composition of biochars was determined using an elemental analyzer (Vario EL Cube, Langenselbold, Germany). The bulk element composition of O was calculated using the difference of unity and C, N, H, S, and ash (ash was measured by burning biochar in a muffle at 800 °C for 4 h). Fourier transform infrared spectroscopy (FTIR) was used (Nexus-670, Nicolet, Massachusetts, MA, USA) and recorded in the region of 400–4000 cm^−1^. The resolution was set as 1.0 cm^−1^. X-ray photoelectron spectroscopy (XPS) was conducted with an Escalab 250Xi (Thermo Fisher, Waltham, MA, USA), using focused monochromatized Al Kα radiation (hν = 1486.6 eV). Scanning electron microscopy and energy-dispersive spectroscopy (SEM-EDS) were carried out with Nova NanoSEM 450 (FEI, Hillsboro, OR, USA) and AZtec X-MaxN 80 (Oxford, UK). Beckman Coulter SA3100 (Beckman Coulter, Chaska, MN, USA) was used to test the specific surface area and porosity of the biochar samples.

## 3. Results and Discussion

### 3.1. Comparison of Bulk and Surface Element Composition

Table 1 describes the compositional changes during oxidation. The data of the bulk element composition were obtained from the elemental analyzer, and data of the surface composition were obtained from XPS. Higher N values have been obtained by both techniques, XPS and elemental analysis, for the biochar that were oxidized with HNO_3_/H_2_SO_4_, probably due to the generation of N surface groups in form of NO_2_-C. C was the most abundant component in biochar. From the table, it can be seen that C decreased up to 22.4% in PRSB in pretreatment, whereas only 0.06% decreased in PRB. This indicates that biochar produced from reed contained less soluble organic carbon. The C content of AWSB, ARSB, and ARB reduced by 17.8%, 32.0%, and 24.9%, respectively, compared to the original biochars in treated with acid oxidation, and the O content increased by 585.1%, 1431.3%, and 636.5%, respectively. Basic oxidation showed similar changes: the C content of BWSB, BRSB, and BRB reduced by 7.9%, 11.5%, and 4.2%, respectively, and the O content improved by 286.2%, 610.0%, and 139.0%, respectively. The increased O content was provided by oxygen-containing functional groups as mentioned in Section 3.2. O/C is thought to be related to hydrophilicity [18]. In this study, the acid-oxidation treated biochar showed higher O/C than that of basic oxidation. O/C in AWSB, ARSB, and ARB increased by 825.0%, 1866.7%, and 1000.0%, respectively, compared to the original biochars. The great improvement in O/C augmented the hydrophilicity of acid-treated biochar. Hence, appropriate oxidation of biochar in the field can increase the interstitial water and moisture content of the soil. There was almost 2.3%, 0.5%, and 0.6% carbon reduction in PWSB, PRSB, and PRB. Carbon loss on the surface of PWSB was much lower than the bulk, indicating that less water-soluble carbon was contained in the surface of wheat straw-derived biochar.

Great differences were observed between the bulk element composition and surface element composition. The percentage of carbon was higher on the surface, and in general, there was less oxygen. In a former study [16], we thought oxidation formed an aging layer on the surface of biochar and observed great differences between the bulk element composition and surface element composition. Through several analysis methods, we concluded that the aging layer was characterized by high C% and O/C. This study can confirm our former results. In addition, we found that the aging layer varied among different materials. Biochars produced from reed had more persistent carbon than biochar from wheat straw and rice straw as a result of less carbon loss on the surface of reed-derived biochar.

### 3.2. Oxygen-Containing Functional Groups on the Surface

**Figure 1:**
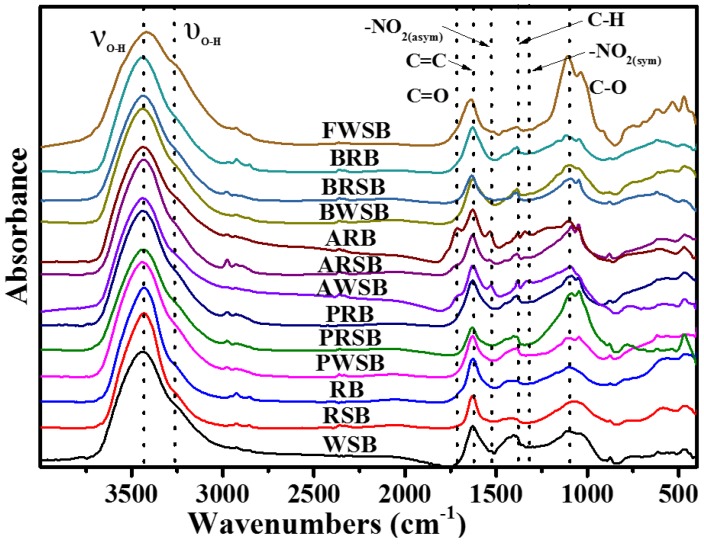
Fourier transform infrared spectroscopy of biochars from different processes.

**Figure 2:**
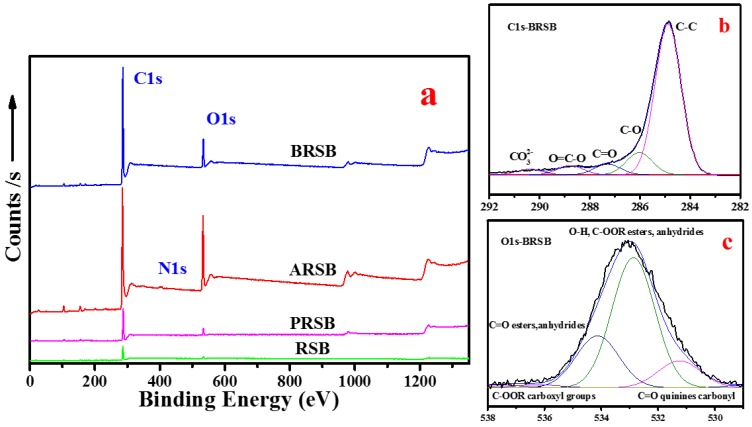
(**a**) XPS curves from treated reed-derived biochars and (**b**,**c**) PeakFits of BRSB.

FTIR and XPS were conducted and results are shown in Figure 1 and Figure 2. From Figure 1, all biochars showed similar absorption, though they were produced from different materials. There were four main absorption bands, the results are as follows: the –OH stretching vibration (ν_O–H_, 3400 cm^−1^), the stretching vibration of –C=C– (C=C, 1600 cm^−1^), the deforming vibration of C–H (C–H, 1395–1370 cm^−1^), and the stretching vibration of C–O (C–O, 1260 cm^−1^). Meanwhile, four weak absorption bands also attracted our attention, and they were υ_O-H_ stretching at 3300–3200 cm^−1^, C=O stretching at 1700 cm^−1^, asymmetric –NO_2_ stretching at 1615–1510 cm^−1^, and symmetric –NO_2_ stretching at 1390–1320 cm^−1^.

C–O had the most observed change in the four main absorption bands; the others did not have significant changes. Acid-treated biochar showed a great increase in the 1710 cm^−1^ absorption band (C=O), which was thought to be related to carboxyl functional groups or the aromatic ring [19]. Two absorption bands appeared in the acid-oxidation treated biochar only, asymmetric –NO_2_ (1615–1510 cm^−1^) and symmetric –NO_2_ (1390–1320 cm^−1^). They were nitro groups formed by HNO_3_ and H_2_SO_4_ in the carbon matrix. In general, oxygen-containing functional groups increased in all treated biochars, especially in that treated with acid oxidation.

From the FTIR results, more oxygen-containing functional groups were found in oxidized biochars, so we performed XPS for further study of carbon and oxygen, and the results are given in Figure 2. A great increase in C1s can be observed in the curves, indicating that functional groups were growing under oxidation. O1s increased from 0.5% (RSB) to 19.3% (ARSB). A weak N1s peak appeared in acid-treated biochar. C1s and O1s in XPS were conducted in PeakFit (Systat PeakFit, Version 4.12, Seasolve, San Jose, CA, USA) to study their details. C1s can be divided into five peaks: C–C at 284.8 eV, C–O at 285.7 eV, C=O at 287.0 eV, O=C–O at 288.9 eV, and CO_3_^2−^ at 289.4 eV. O1s was divided into four peaks: C=O from carbonyl at 529.8 eV, O–H or C–OOR from esters and anhydrides at 531.5 eV, C=O from esters or anhydrides at 533.1 eV, and C–OOR from carboxyl groups at 535.0 eV [20]. The integral area normalization method was used to study the relative ratio of each peak in C1s and O1s, and results are shown in Table 2. From C1s PeakFit results, all the oxygen-containing functional groups except CO_3_^2−^ increased in all pretreated, acid, and basic processes for carbonate decomposed into CO_2_ and H_2_O. Carboxyls were found to be increased in O1s PeakFit.

### 3.3. Surface Structure of Biochars

**Figure 3:**
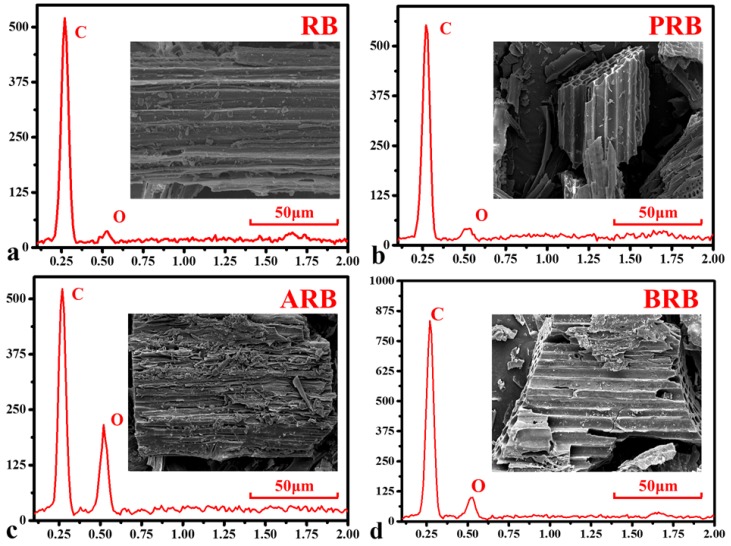
(**a**) SEM and EDS of reed-derived biochar; (**b**) SEM and EDS of pretreated reed-derived biochar; (**c**) SEM and EDS of acid treated reed-derived biochar; (**d**) SEM and EDS of basic treated reed-derived biochar.

The results of SEM-EDS are given in Figure 3. From SEM, a few chippings were interspersed in ditches on the surface of RB, and most of them were washed down in PRB. Ditches in RB, PRB, and BRB were complete and clean, but destroyed ditches and scars were observed in ARB because of the decomposition of unstable substances, such as carbonates. BRB showed similar changes but to a weaker extent than ARB. EDS indicated that O/C increased by 12.5%, 252.1%, and 96.9% for PRB, ARB, and BRB, respectively. Most of the increased O appeared as oxygen-containing functional groups. A high O/C led to more hydrophilicity of the biochar; meanwhile, the addition of oxygen on the surface improved complexation between metal ions and functional groups. Some other SEM-EDS results are shown in Supplementary Figure S1.

**Figure 4:**
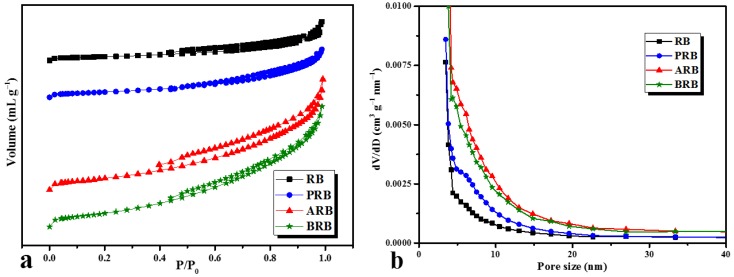
(**a**) N_2_ adsorption–desorption isotherms and (**b**) pore size distribution of RB, PRB, ARB, and BRB.

The destroyed ditches and scars increased the surface porosity of the biochar. From Figure 4, all biochars exhibited a type IV isotherm and H4 hysteresis loop, indicating a considerable mesopore structure on the surface. The specific surface areas of RB, PRB, ARB, and BRB were 11.265 sq.m g^−1^, 13.642 sq.m g^−1^, 29.840 sq.m g^−1^, and 18.362 sq.m g^−1^, respectively, from surface analysis. The surface area of PRB, ARB, and BRB increased by 21.1%, 164.9%, and 63.0%, respectively. The growth in specific surface area and porosity increased the physical adsorption of biochar, which improves the physiochemical properties, such as the water-holding capacity. The N_2_ adsorption–desorption isotherms of wheat and rice straw-derived biochars are given in Supplementary Figure S2.

### 3.4. Effects of the Aging Process on Biochar Adsorption

**Figure 5:**
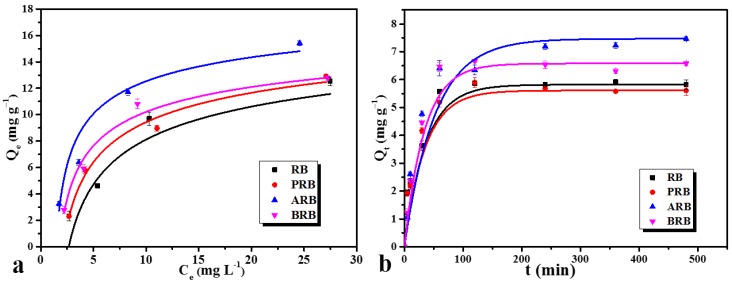
(**a**) Adsorption isotherms and (**b**) kinetics of RB, PRB, ARB, and BRB.

Figure 5 describes the adsorption isotherms and kinetics of RB, PRB, ARB, and BRB. To evaluate the adsorption capacity, kinetic models (pseudo-first-order and pseudo-second-order adsorption) and isotherm models (Langmuir isotherm and Freundlich isotherm) were used to correspond with the adsorption data, and the parameters of models are shown in Table 3. From the results, the pseudo-second-order model is more suitable for Cd adsorption than the pseudo-first-order model, which means the adsorption of Cd on biochar is a complex process (containing both physical and chemical reactions). From the kinetic curves, the adsorption capacity of ARB increased by 28.4% compared with RB, and the absorption capacity of BRB increased by 13.1%, whereas a slight decrease was found in PRB. The adsorption reached saturated in 120 min.

The kinetics model proved that the adsorption of Cd on biochar is the result of both physical and chemical reactions. From the above analysis, the increased porosity and functional groups were the main reasons for the increased adsorption capacity. During oxidation, the surface structure was destroyed and the ditches and scars enhanced electrostatic attraction. FTIR and XPS showed that many more functional groups were found on the surface of oxidized biochar, and these functional groups combined with Cd^2+^ by complexation. Meanwhile, the Langmuir model was more suitable for isotherms, indicating that adsorption is a process of monolayer interaction involving energetically equivalent independent sites [9]. The adsorption isotherms and kinetics of other biochars are given in Supplementary Figure S3 and they also corresponded with the pseudo-second-order and Langmuir models very well. However, the adsorption capacity of wheat and rice straw-derived biochar was weaker than that of reed-derived biochar, which could be because the inorganic compounds in wheat and rice straw-derived biochar contended with Cd for the adsorption sites.

### 3.5. Aging Process of Biochar in the Field

FTIR of FWSB showed some differences between simulated oxidation and the field aging process. In FWSB, much greater peaks were observed in C–O and O–H, indicating an increase in alcohols, aldehydes, or other related functional groups. All C=C peaks, in both simulated oxidation and the field aging process, had stable percentages in FTIR, which means aromatics did not have significant changes on the surface. We also found a weak peak in –NO_2_, and previous studies indicate that acid rain was responsible for this.

**Figure 6:**
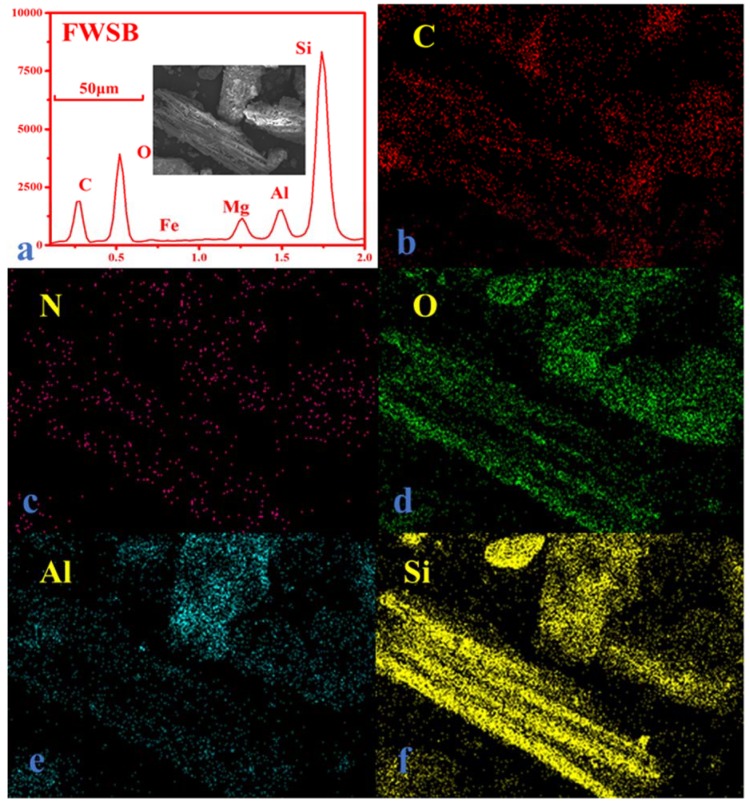
(**a**) SEM and EDS of FWSB; (**b**) C mapping of FWSB; (**c**) N mapping of FWSB; (**d**) O mapping of FWSB; (**e**) Al mapping of FWSB; (**f**) Si mapping of FWSB.

Figure 6 shows the SEM and EDS of FWSB. Ditches on the surface were destroyed, and many pores were filled with minerals. From the EDS curve, the aging process (in soil for eight years) significantly increased the percent of O and led to an improvement in O/C on the surface. Compared with WSB, O/C in EDS increased by 2333% in FWSB. The increased O not only increased the hydrophilicity of biochar, but increased the complexation as well. However, oxygen, which is part of the existing minerals in soil, may also contribute to the increase in O content; further studies are needed to investigate this. The most significant changes were found in the mineral contents. Not many minerals were observed in simulated oxidation, but from mappings of FWSB, Si was scattered all around the biochar surface and had the most atomic percentage on the surface. Others, like Fe, Mg, and Al, also accounted for a large proportion. Some of them were complexed with functional groups in oxides, and some may have been adsorbed by physical attraction. Therefore, biochar may do a good job when applied to soil contaminated with heavy metals to prevent heavy metal ions from getting into crops.

## 4. Conclusions

In this study, two oxidation methods were used to simulate different aging processes of biochar in soil. The results show that aged biochars were equipped with an aging layer on the surface, and the characteristics of the aging layer varied in different materials. The aging layer of reed-derived biochar contained more persistent carbon than wheat straw and rice straw-derived biochar. More oxygen-containing functional groups were found in aged biochar, such as carboxyl. Meanwhile, nitro functional groups appeared in the acid-oxidation treated biochar. Destroyed ditches and scars were observed in aged biochar, which was responsible for the increase in specific surface area and surface porosity. More aliphatics and minerals were found on the surface of field-aged biochar. An increased porosity and the presence of functional groups was the main reason for the improved adsorption capacity of oxidized biochar.

## Figures and Tables

**Table 1:** Element composition of biochars.

Sample	Bulk Element Composition (%)							Surface Element Composition (%)			
	N	C	H	S	Ash	O	O/C	N	C	O	O/C
WSB	1.12	65.75	1.41	0.44	28.39	2.89	0.04	0.87	91.92	7.21	0.08
RSB	1.31	68.15	2.09	0.51	26.15	1.79	0.03	0.02	87.25	0.05	0.00
RB	1.22	69.27	3.15	0.38	22.88	3.1	0.04	0.84	90.38	0.12	0.00
PWSB	1.42	56.38	1.98	0.33	25.93	7.96	0.13	1.45	89.83	8.38	0.09
PRSB	1.06	52.90	1.41	0.41	25.04	9.18	0.15	0.01	86.78	0.05	0.00
PRB	1.23	69.23	3.34	0.27	21.46	4.47	0.06	0.95	89.88	0.31	0.00
AWSB	2.84	54.06	1.84	0.74	20.72	19.8	0.37	3.38	78.91	16.64	0.21
ARSB	1.51	46.33	1.58	1.61	21.56	27.41	0.59	2.55	77.21	19.3	0.25
ARB	3.50	52.01	2.58	0.67	18.41	22.83	0.44	1.07	90.18	8.75	0.10
BWSB	1.47	60.57	1.60	0.93	24.27	11.16	0.18	1.92	85.65	10.91	0.13
BRSB	1.35	60.28	1.44	0.64	23.58	12.71	0.21	0.91	84.19	6.35	0.08
BRB	1.72	66.33	3.10	0.38	21.06	7.41	0.11	0.57	90.23	3.69	0.04

Bulk element compositions were obtained from elemental analyzer, and surface element composition from XPS. WSB, wheat straw biochar; RSB, rice straw biochar; RB, reed biochar; PWSB/PRSB/PRB, pretreated WSB/RSB/RB; AWSB/ARSB/ARB, acid WSB/RSB/RB; BWSB/BRSB/BRB, basic WSB/RSB/RB.

**Table 2:** Relative ratio of peaks in C1s and O1s from XPS.

Sample	C1s					O1s			
	C–C	C–O	C=O	O=C–O	CO_3_^2−^	C=O (Carbonyl)	O–H (Esters)	C=O (Esters)	C–OOR (Carboxyl)
WSB	0.72	0.12	0.07	0.05	0.04	0.12	0.62	0.24	0.02
RSB	0.75	0.1	0.07	0.05	0.03	0.09	0.65	0.22	0.04
RB	0.78	0.11	0.06	0.03	0.02	0.13	0.65	0.2	0.02
PWSB	0.7	0.12	0.08	0.06	0.04	0.07	0.68	0.2	0.05
PRSB	0.74	0.1	0.07	0.06	0.03	0.09	0.65	0.21	0.05
PRB	0.76	0.12	0.07	0.03	0.02	0.15	0.64	0.17	0.04
AWSB	0.65	0.16	0.09	0.09	0.01	0.16	0.59	0.19	0.06
ARSB	0.69	0.14	0.08	0.07	0.02	0.14	0.6	0.18	0.08
ARB	0.69	0.13	0.09	0.08	0.01	0.14	0.59	0.2	0.07
BWSB	0.69	0.13	0.07	0.08	0.03	0.14	0.61	0.21	0.04
BRSB	0.74	0.11	0.07	0.06	0.02	0.13	0.62	0.2	0.05
BRB	0.76	0.11	0.08	0.04	0.01	0.13	0.67	0.18	0.02

**Table 3:** Parameters of pseudo-first-order, pseudo-second-order, Langmuir isotherm, and Freundlich isotherm models.

Sample	Pseudo-First-Order		Pseudo-Second-Order		Langmuir Isotherm			Freundlich Isotherm		
	*K*_1_	*R* ^2^	*K*_2_	*R* ^2^	*Q*_max_ (mg g^−1^)	*K*_L_	*R* ^2^	*n*	*K*_F_	*R* ^2^
WSB	0.026	0.903	0.031	0.958	5.57	0.25	0.92	2.93	1.51	0.91
PWSB	0.026	0.827	0.038	0.940	5.65	0.25	0.91	3.01	1.38	0.85
AWSB	0.027	0.925	0.032	0.972	6.31	0.24	0.99	2.64	1.73	0.93
BWSB	0.026	0.909	0.036	0.955	6.02	0.26	0.94	2.13	2.06	0.83
RSB	0.028	0.894	0.032	0.942	5.52	0.24	0.94	3.18	1.43	0.87
PRSB	0.028	0.882	0.036	0.902	6.04	0.27	0.92	2.85	1.61	0.85
ARSB	0.027	0.852	0.033	0.920	6.74	0.25	0.97	3.24	1.27	0.92
BRSB	0.028	0.942	0.037	0.974	6.21	0.26	0.87	3.06	1.34	0.89
RB	0.028	0.974	0.035	0.984	6.17	0.23	0.94	3.14	1.44	0.91
PRB	0.027	0.852	0.037	0.951	6.15	0.24	0.98	3.30	1.28	0.95
ARB	0.027	0.913	0.031	0.924	7.52	0.27	0.96	2.04	3.13	0.93
BRB	0.026	0.920	0.033	0.963	6.60	0.24	0.98	3.06	1.70	0.94

## Supplementary Materials

The following are available online at http://www.mdpi.com/1996-1944/11/12/2362/s1, Figure S1: SEM and EDS of RSB, PRSB, ARSB and BRSB, Figure S2: N_2_ adsorption-desorption isotherms of wheat (a) and rice (b) straw derived biochars, Figure S3: Adsorption isotherms and kinetics of rice straw derived biochars (a,b) and Wheat straw derived biochars (c,d).
