# Supplementary material for: Effects of Wet Oxidation Process on Biochar Surface in Acid and Alkaline Soil Environments
Source: Materials (Basel). 2018 Nov 23;11(12):2362. doi: 10.3390/ma11122362 (PMC6317012; doi:10.3390/ma11122362)
Supplement: Supplementary file 1 [file materials-11-02362-s001.docx]

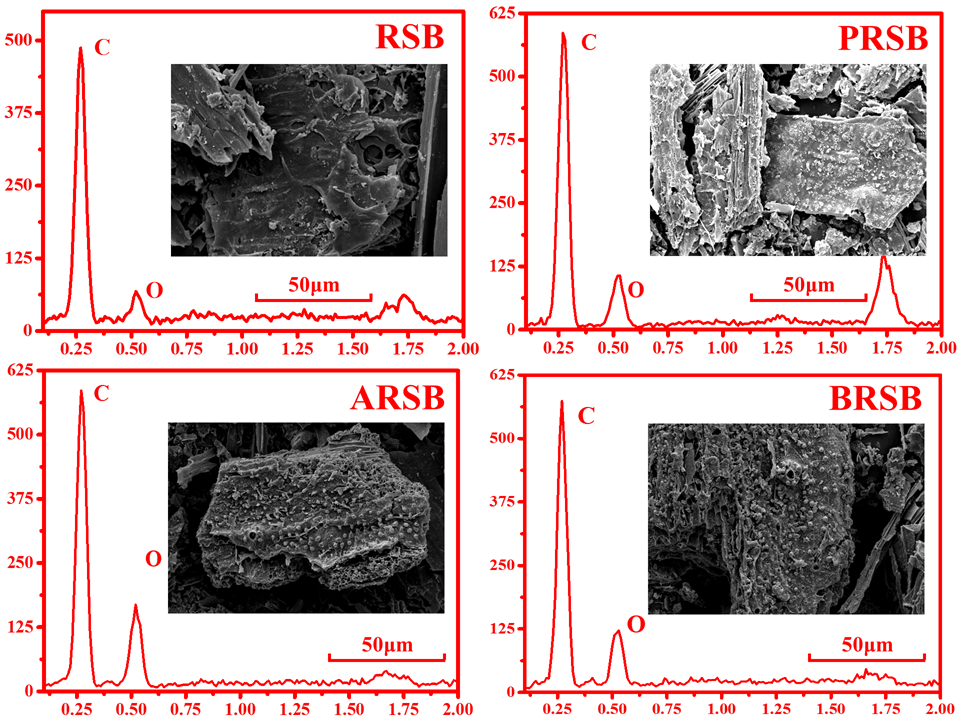


**Figure S1.** SEM and EDS of RSB, PRSB, ARSB and BRSB.


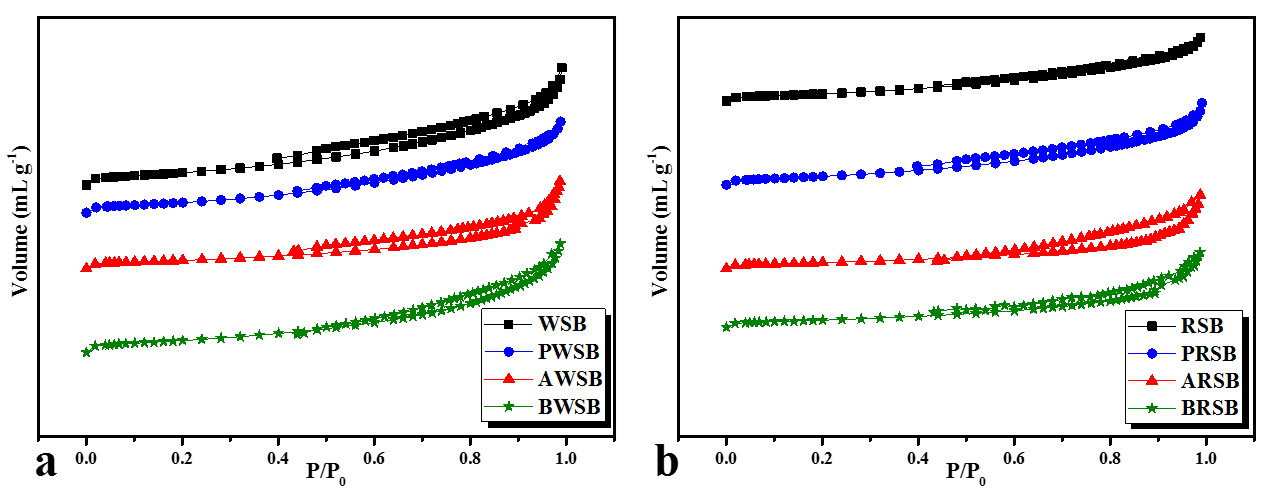


**Figure S2.** N_2_ adsorption-desorption isotherms of wheat (**a**) and rice (**b**) straw derived biochars.


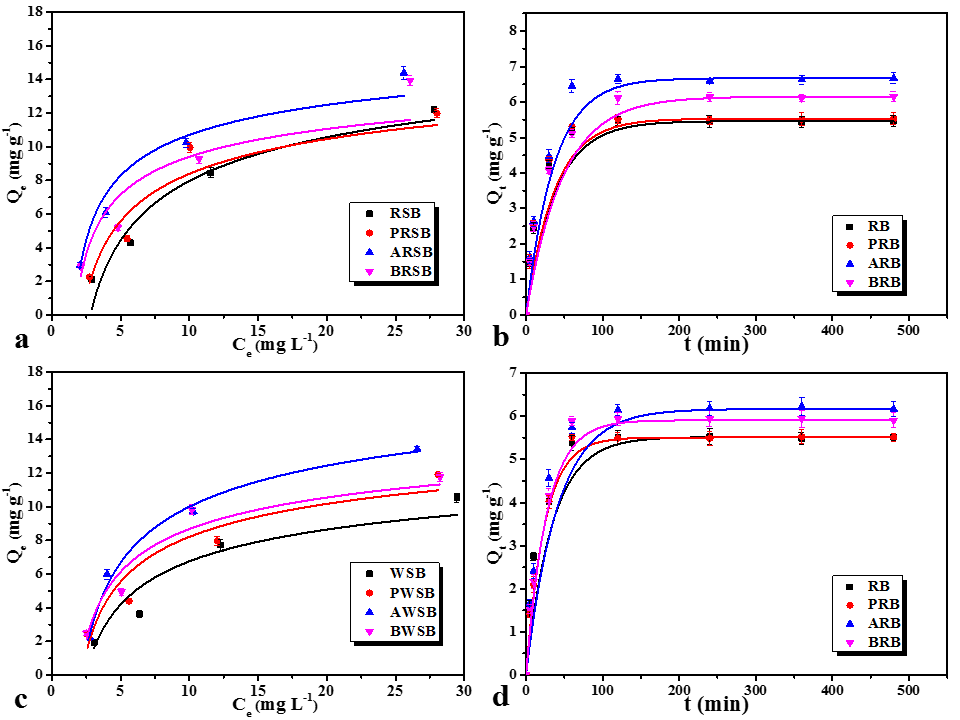


**Figure S3.** Adsorption isotherms and kinetics of rice straw derived biochars (**a**,**b**) and Wheat straw derived biochars (**c**,**d**).
